# Why Should We Study the Foreign Language Effect: Debiasing through Affecting Metacognition?

**DOI:** 10.3390/jintelligence11060103

**Published:** 2023-05-30

**Authors:** Michał Białek

**Affiliations:** Psychology of Management Unit, Institute of Psychology, University of Wroclaw, 50-527 Wrocław, Poland; michal.bialek3@uwr.edu.pl

**Keywords:** intuition, reflection, metacognition, debiasing, foreign language effect

## Abstract

Debiasing is a method of improving people’s decisions by reducing their reliance on salient intuitions causing them to behave suboptimally or biasedly. However, many of the known debiasing techniques have limited effectiveness or can only remedy a one-shot decision, rather than having a lasting impact. In this work, I focus on the role of metacognition in debiasing decision-making and discuss how it can be better understood through the lens of the foreign language effect. The foreign language effect suggests that using a foreign language can sometimes benefit people’s decision-making without providing them with additional information or instructions regarding the task. However, we do not fully understand how the foreign language effect works and its limitations. I conclude by urging scientists to research this effect with the hope of having a lasting positive impact on society.

## 1. Introduction

Following the 9/11 World Trade Center attack, the United States established an “enhanced interrogation program” in detention camps such as Guantanamo Bay with the intention of preventing future terrorist attacks. However, as early as three months after the program was launched, reports suggested that techniques such as waterboarding and sleep deprivation were ineffective in preventing attacks (Senate Select Committee on Intelligence 2012). Despite this, the program persisted for over a decade. In hindsight, continuing (and even starting) the program was a bad decision.

There are many possible explanations for why people make bad decisions that, in hindsight, appear to be obviously incorrect. Some errors are simply random noise, as people decide by sampling from their internal distribution of values (Stewart et al. 2006), or by selectively assigning queries to internalized preferences (Johnson et al. 2007). In other words, people do not have a fixed set of preferences but rather decide ad hoc. Our preferences are not fixed parameters (e.g., 7 out of 10), but rather a distribution of parameters, in which some values are more probable than others, but many are possible nonetheless. To illustrate, assume you have developed a taste for lasagna, but you may wish to eat it more or less strongly on a given day. Decisions are being made similarly, and even experts make mistakes due to the natural “noisiness” of human cognition (Kahneman et al. 2022). However, some errors are predictable, to the extent that for some type of problems most people tend to commit the same error (Kahneman et al. 1982). Decision errors, such as starting the torture program by the US government, may occur because decision-makers lack the required knowledge or expertise to assess all arguments and potential consequences of their decision (Larrick and Feiler 2015). People may also lack the cognitive abilities to process all the available information (Maran et al. 2020). Finally, some people may be blinded by their beliefs and ignore inconsistent information (Kunda 1990).

However, there is something potentially more profound and often overlooked in the literature that contributes to imperfect decision-making—metacognition, that is thinking about thinking (Dunlosky and Metcalfe 2008). By allowing the decision-maker to take an outside perspective and reevaluate their thinking, metacognition can improve decision-making. It helps an individual to monitor their cognition, resulting in subjective feeling of its quality and accuracy—confidence or feeling for rightness (Ackerman and Thompson 2017; Thompson et al. 2011). If the confidence is low, an individual allocates more cognitive resources into the ongoing cognitive processing, which in turn is likely to improve its quality and accuracy. In this essay, I will explore the functions of metacognition and examine whether it can be facilitated or distorted.

Metacognition is quite different from cognitive abilities, such as intelligence, which are responsible for information processing. The best illustration of the difference between cognitive abilities and metacognition in the context of decision-making I can think of is chess. When you try to solve a chess puzzle, you know there is a winning move. All you have to do is to consider all the possible moves and their follow-ups and see which of these combinations leads to a win. This is a lot of information you must process, and the outcome of your thinking depends on your cognitive abilities. However, things are far more complicated in a real-life competitive chess game for which you have limited time. You can lose a game when having a worse position and ending up being checkmated, but you can also lose on time. Hence, you cannot go into such deep deliberation as in a chess puzzle for every move. To manage your time efficiently, you have to know when to reflect and search for this single good move, and when no deep thinking is needed because there are several equally good moves and you can select any of them without much impact on the game. Deciding when to think depends on metacognition. This may refer to thinking before deciding, but also to cases when one has to re-evaluate a decision that has already been made. 

The torture program of the USA I described in this work illustrates a failure of metacognition—the error stems from decision-makers not realizing they must stop and think, reevaluating all available evidence. They may have been operating under the assumption that these techniques were necessary for obtaining valuable intelligence and protecting national security. However, they may not have taken the time to critically evaluate the effectiveness of these methods, or to consider the ethical and moral implications of using such methods. Additionally, they may not have reflected on the potential long-term consequences of using these techniques, such as damage to the reputation of the country, legal challenges, or creating more radicalized individuals. In this scenario, the lack of metacognitive insight—the ability to step back and reflect on one’s own thought process—led to a decision that was not fully informed and not considering the broader context and implications. Even after learning the results of the program were not as expected, the decision-makers failed to stop and reevaluate the decision they once made. This example illustrates that by going forward and not reflecting on their intuitions and past decisions, even the most gifted person will fail at a task because they have not even started thinking. Without metacognitive insight, whatever made these certainly intelligent individuals decide in a particular way will never be corrected, in turn escalating commitment to a bad decision, and risking them repeating the same decision error in the future. 

As mentioned earlier, a failure of metacognition can occur when an individual fails to reflect on their own thinking. However, there is another less obvious but equally problematic failure of metacognition: overthinking. For example, a person may be spending too much time analyzing and comparing options that are only slightly different from each other. This inefficient allocation can result in a waste of time and resources for both the individual and those involved in the decision-making process. Effective metacognition, on the other hand, helps individuals allocate their cognitive resources efficiently by directing their attention toward the most important and relevant tasks.

Hopefully, it is becoming clear that metacognition, or thinking about our own thinking, is crucial for good decision-making. It helps decision-makers become aware of their internal processes, the cues they use, and to monitor and calibrate their confidence in their decisions (Ackerman and Thompson 2017). In this work, we are going to look into metacognition more closely. We will learn about what it is and how it works, and we will see if we can find ways to make it better. Specifically, I will present research on the foreign language effect, which suggests that using a non-native language can influence metacognitive processes and enhance certain types of decision-making, such as risky choices (Circi et al. 2021; Stankovic et al. 2022). My ultimate goal is to determine if the insights gained from studying the foreign language effect and metacognition can be applied to improve everyday decision-making. However, while we can artificially boost metacognition in experimental settings, it is not always beneficial, and we still do not fully understand why it works and when it will be effective. Therefore, although the foreign language effect research may not provide an immediate solution to decision-making difficulties, I believe that the long-term potential of this research is promising. 

This essay is intended as an introductory reading to help you familiarize yourself with the topics of debiasing, metacognition, and the foreign language effect. I will discuss basic concepts and current directions in debiasing research. In conclusion, after examining other existing explanations of the effect and finding inconsistent support, I propose a working hypothesis: the use of a foreign language distorts our metacognitive processing, leading us to reflect more on problems that have intuitively appealing but incorrect solutions. I suggest that people become less sensitive to internal cues indicating the correctness of their intuitive responses. Consequently, they allocate cognitive effort more randomly, occasionally reflecting on problems that elicit highly appealing intuitions. This approach helps individuals avoid relying on incorrect but intuitively appealing responses and instead discover less obvious yet normatively correct solutions, resulting in improved performance but potentially unnecessary reflection on simple problems. In this model, improved judgment and decision-making is merely a side-effect of distorted metacognition. However, this is only a working hypothesis, supported by limited evidence, mostly coming from my own research.

## 2. How to Know When to Think?

As I argued before, much of our thinking relies on mental shortcuts, preserving many of our cognitive resources at the cost of slightly reduced accuracy. These shortcuts, or heuristics, are typically well-calibrated and produce accurate and satisfying solutions (Gigerenzer 2000). The problem with thinking is that heuristics are not always accurate, but one cannot always verify them because that would be burdensome (De Neys 2022; Stanovich 2020). Hence, metacognition has the duty to monitor our thinking and intervene when needed by triggering reflective processes.

Some insight into the question of how people know when to stop and reflect can be gained from a line of research started by Wim De Neys. He presented people with tasks that have salient incorrect intuitions (which he called conflict tasks) and tasks in which such an intuition is correct (no-conflict tasks). He then compared intuition-based responses in these two types of tasks, finding that people are less confident, memorize more details of a task, and require a longer time to provide an intuition-based response in conflict tasks than in co-conflict tasks (De Neys and Glumicic 2008). Brain areas responsible for response inhibition (i.e., lateral prefrontal cortex) were activated in cases when a person responded correctly to a conflict task, but also when responding incorrectly, suggesting each time a person had to inhibit one of their considered responses (De Neys et al. 2008). Hence, even though a person responded incorrectly to a conflict task, they still suffered internal conflict between their gut feelings and a normatively correct response. This metacognitive experience of internal conflict is claimed to trigger reflection allowing one to decouple and verify the intuitive response (De Neys and Pennycook 2019; Pennycook et al. 2015)^1^. 

Individuals differ in their sensitivity to metacognitive cues. Typically, metacognition is inferred from confidence ratings and their relationship to the response accuracy (Fleming and Lau 2014). Here, one is expected to show higher confidence in own responses when they are correct compared to when they are wrong, or, to put this differently, confidence should be strongly positively correlated to response accuracy. Another instance of faulty metacognition is the Dunning-Kruger effect, in which poor performers overestimate their performance, while top performers sometimes underestimate their performance (Kruger and Dunning 1999). An alternative and extremely popular method used to measure metacognitive sensitivity is the Cognitive Reflection Test (Frederick 2005)^2^. In its original version, the test consists of just three items. One of them is the famous bat-and-ball problem:

*A baseball bat and a ball cost $1.10 together, and the bat costs $1.00 more than the ball, how much does the ball cost?*

Oftentimes, people reply the ball costs 10 cents, which is incorrect. If a ball would cost 10 cents, and the bat $1.00, then the two items would indeed cost together $1.10, but the difference between the two items would be only 90 cents rather than $1.00. What is most surprising, people seem to not improve in this task after multiple exposures—their performance is constant (Stagnaro et al. 2018), and it correlates similarly with other individual differences or decision performance in naïve and experienced participants (Białek and Pennycook 2018). In other words, people who provide an incorrect response are typically so satisfied that they never reinvestigate it (Raoelison and De Neys 2019). 

Cognitive reflection is a crucial aspect of effective decision-making. Individuals who are more cognitively reflective tend to display a number of positive attributes such as less steep discounting (Frederick 2005), a reduced susceptibility to decision frames in the discounting problem (Białek and Sawicki 2018), a greater ability to differentiate between true and false news (Pennycook and Rand 2019), less overconfidence (Białek and Domurat 2018), and fewer dogmatic beliefs and paranormal beliefs (Pennycook et al. 2012). Surprisingly, the benefits of cognitive reflection are independent of their cognitive abilities (Stanovich and West 2008). Differently put, without cognitive reflection the benefits of high cognitive abilities are much smaller (but smarter people may have more accurate intuitions, Raoelison et al. 2020).

Finally, people’s metacognitive sensitivity to a conflict seems stable over time. In a study by Voudouri et al. (2022), participants answered a set of heuristic-and-bias tasks quickly and under cognitive load, and then were given an opportunity to reflect and change their response (a so-called two-response paradigm, Thompson et al. 2011). This task was then administered to them again two weeks later, again using the same two-response procedure. The majority of people consistently provided the same response, i.e., they either responded correctly or incorrectly in the intuitive and reflective stages of the experiment. Those who were more conflicted in the intuitive stage tended to change their responses more eagerly when given an opportunity to further reflect on a problem in the same session and after repeating the same task after several weeks. Critically, people were more likely to change their responses in conflict rather than no-conflict problems, again suggesting that they detect the intuition-based response they provided may be incorrect. That said, even those who answer incorrectly tend to be subtly aware of the fact that their reasoning may be biased. They use this metacognitive information to engage in reflection given the opportunity, and in turn, are more likely to change their response. 

## 3. Can Metacognition Be Improved?

Above, I argued that metacognitive sensitivity to cues (i.e., cognitive reflection) is critical for good decision-making because it can trigger reflection to (1) correct an intuitive response and (2) allow for a response change given the opportunity to make the same decision a second time. Since people differ in their metacognitive sensitivity, a question arises whether this trait is fixed or can be improved. I already reviewed some studies that showed that cognitive reflection seems to be relatively stable across time and situations. Some studies reported increased performance in the Cognitive Reflection Test (CRT) after prior exposure (Haigh 2016; Stieger and Reips 2016). However, such a result is likely an artifact because those who performed well on the CRT are simultaneously more likely (1) to improve and (2) to recall even taking the test (Stagnaro et al. 2018). Hence, when the prior experience with the test is self-reported, it biases the results of the analysis because those who performed well on it are also more likely to recall having taken it.

As I mentioned in the previous subsection, people seem not to gain better insight into a task by merely repeating it. What is more, even providing feedback on accuracy in a conflict task does not seem to help people improve. To illustrate, Janssen et al. (2020) observed that the few who did improve after receiving feedback on the bat-and-ball problem also showed improved conflict detection. That is, it appears that they learned to be sensitive to the logic-based cues and metacognitively realize that these cues conflict with the intuition-based cues. Hence, by learning how a task is designed and how to solve it, some people were able to increase their metacognitive sensitivity to logical intuitions. 

Consistent findings are reported in the research on the Monty Hall problem (Selvin 1975). Here, as in the famous TV show “Let’s Make a Deal”, the setup is as follows: you are a contestant on a game show and you are presented with three doors. Behind one door is a valuable prize (e.g., a car), and behind the other two doors are goats. You choose one door, but before the prize behind that door is revealed, the host, Monty Hall, who knows what is behind the doors, opens one of the other two doors to reveal a goat. Monty then offers you the option to stick with your original choice or to switch to the remaining door. The question is “what should you do to maximize your chances of winning the prize?”

The solution to the Monty Hall problem is that the contestant should switch their choice. The reasoning behind this is that the probability of the prize being behind the original choice is 1/3 since there are three doors and one of them has the prize. However, after Monty opens one of the other doors to reveal a goat, the probability of the prize being behind the remaining closed door is 2/3. This is because the probability of the prize being in any of the unselected doors is 2/3. Since Monty would not open a door if the prize was behind it, and since there are now only two doors remaining, the odds of the prize being behind the unopened door are now 2/3, which is a significant improvement over the 1/3 chance of winning by sticking with the original choice.

The problem is hard and very abstract, and thus it is unsurprising that many people fail to respond correctly to it (Gilovich et al. 1995). 

What is more surprising, people tend to stick with their initial choice (i.e., not to switch doors) even when given the opportunity to learn the true probability of winning for each option. This was shown in an experiment where participants repeatedly solved the problem over 80 trials. Although the decision to switch became more common in later trials, it still occurred in only slightly more than 50% of decisions. Additionally, even with various forms of feedback, participants did not improve their understanding of the task (Saenen et al. 2015). So, as with the Cognitive Reflection Test, people seem to learn very little from their own mistakes. 

My own efforts in improving the Cognitive Reflection Test scores were somewhat more promising: in one line of research, I asked people additional questions about the Cognitive Reflection Test items before asking them the main question. For example, for the bat-and-ball problem, I asked them a question that has a correct response, “What is the most popular sport in the US?”, or an opinion question that has no single correct response, “Name a sport, other than baseball, that can be played with bat and ball”. My idea was that once people start deliberating on a problem, they will subsequently default to that processing mode, but only when reflection would be accuracy-oriented (as in questions but not in opinions). Consistently with my hypothesis, the Cognitive Reflection Test scores improved only when preceded by questions and not by opinions (Białek 2016). However, the effect was borderline significant, observed only in a subsample of naïve participants, and I subsequently failed to replicate it in larger samples from the Amazon Mechanical Turk. 

The second line of research that showed improvement in the Cognitive Reflection Test score was serendipitous: we investigated moral judgments under alcohol intoxication (Paruzel-Czachura et al. 2021). One of the tested ideas was that maybe the effect of alcohol on moral decision-making would be mitigated by high cognitive reflection. The study had a control group that drank orange juice, a placebo group that drank orange juice sprayed with alcohol, and an experimental group that drank orange juice mixed with alcohol. We found that the performance in the CRT improved in the placebo and experimental groups. We believe that people thought that their cognition may have been impaired due to intoxication and were therefore more metacognitively aware of their internal thought processes. Having intensified their metacognitive awareness, they could have spotted the internal conflict between heuristic and logical intuitions and reflected on the problem more than participants in the control group. However, there is a flip side to the coin. It is possible that what we observed is not an improvement in cognitive reflection, but rather, an alteration in the performance on the Cognitive Reflection Test. That is, the results of a psychological test, such as an IQ test, depend not only on the true level of the tested trait but also on the motivation to perform well. To illustrate, people motivated by financial incentives can improve their performance on an intelligence test by 0.64 SD (Duckworth et al. 2011). Differently put, one can appear to gain almost 10 IQ points when motivated properly to perform the test well. Analogously to the effect of incentives on intelligence, believing to be drunk (or being slightly tipsy, as in our study, where BAC ranged from 0.65 to 0.73) could only motivate participants to try harder and thus improve their performance. In both cases (i.e., the intelligence test and the cognitive reflection test) the underlying psychological trait is hypothesized to be constant; what changes is the performance in a test that should measure it. Whichever route the manipulation affected our participants, motivating them to put maximum effort or boosting the cognitive reflection itself, it appears our participants could have reflected slightly more. Yet, it should be stressed again, the effect of alcohol on cognitive reflection is serendipitous, and we worked on replicating it in a preregistered set of experiments.

To conclude this section, it appears that individuals frequently overlook their thinking errors, even when repeating tasks or receiving performance feedback. Although experimental manipulations have shown some promise in enhancing metacognitive awareness, the evidence remains weak and requires further validation.

## 4. The Foreign Language Effect

As a researcher in the field of decision-making, I have observed there are limited psychological interventions that have been robustly shown to improve decision-making outcomes. This is particularly evident in the case of physical exercise, where a large-scale study involving over 60,000 US fitness chain customers found that only approximately half of the 53 interventions tested improved gym participation compared to placebo intervention, and only five interventions performed better than the baseline intervention of text-message reminders and small financial incentives for attendance (Milkman et al. 2021). However, a study by Keysar et al. (2012) caught my attention. The study found that bilingual participants who used their foreign language were less susceptible to cognitive biases compared to those who used their native language. This finding, if robust, has important implications as it suggests that the use of a foreign language can be used as a tool to improve decision-making^3^. This research on the “foreign language effect” on decision-making has been a rapidly growing field and many researchers are excited to see the potential practical applications that could arise from it (McFarlane et al. 2020). 

Some promising research suggested people’s risky choices are better when using a foreign language, with bilinguals showing to be less affected by the framing effect, that is, to prefer risk when the outcomes are described as losses or gains (Costa et al. 2014; Winskel et al. 2016). Moreover, their risky choices were more strategic, with people who decided in their foreign language taking more risks when a lottery was beneficial and taking less risk when the bet was unbeneficial (Hayakawa et al. 2019). What this means is that people using their foreign language take more risks when the expected value of the gamble is positive. Expected value is a multiplication of the potential payoff and its probability, e.g., a 50% chance to win $10 is EV = 0.5 × 10 = 5. A positive gamble is when the EV of a gamble is higher than a certain gain, e.g., of $4. Yet, given such a choice, most people tend to opt for certain smaller gains, displaying so-called risk aversion (Pratt 1964). Since people using their foreign language gambled more often in such positive-EV gambles, using a foreign language is therefore suggested to reduce this aversion (Circi et al. 2021). 

Another risk-related benefit of using a foreign language is that participants were observed to be less affected by the hot hand fallacy when gambling (Gao et al. 2015). The hot hand fallacy is the belief that a person who has experienced success in a particular activity has a higher chance of continued success in the immediate future (Gilovich et al. 1985). In other words, the belief is that a player who has just made a few successful shots in basketball is more likely to make the next shot. However, statistical analysis has shown that this belief is actually a fallacy, as past success does not increase the probability of future success in subsequent activities.

As the research on the foreign language effect progresses, the effect appears more complex and malleable than initially hoped. For example, we found no foreign language effect on risky choice (Borkowska et al. 2023; Muda et al. 2020) and using a foreign language has been shown to distort the ability to delay gratifications (Białek et al. 2022), reason logically (Białek et al. 2020) or the ability to discern true from false news (Muda et al. 2023). Numerous studies from other labs are also reporting no effects of using a foreign language on decision-making. For example, the performance in various heuristic-and-biases tasks was unaffected by the language used (Mækelæ and Pfuhl 2019; Vives et al. 2018). The inconsistencies in the findings can be seen as a positive development, allowing us to better understand the boundary conditions of the foreign language effect, and explain when using a foreign language will affect decision-making, whether the effect will be beneficial, and in turn, whether we can adopt some of this knowledge to debiasing overall, not to bilinguals only.

## 5. The Mechanisms of the Foreign Language Effect

All of this is observed in proficient bilinguals, so it is hard to argue that the foreign language effect occurs because participants do not understand the problem enough to fall into a trap the task is designed to set. Therefore, there has to be a specific mechanism that explains the effect. Understanding how people using a foreign language decide better can lead researchers to propose debiasing techniques that would be much more effective than what is currently proposed. The most promising mechanism, given the current state of the knowledge, is that people who use their foreign language have different access to their metacognitive processes. However, let us discuss the three initial mechanisms through which using a foreign language was supposed to work first (Hayakawa et al. 2016). 

The first speculation was that people using their foreign language experience lower affective arousal than those using their native language. Dedicated studies showed that although people using their foreign language are sometimes less aroused by the problem they face, this change in arousal was not predicting the decision change (Geipel et al. 2015b; Hadjichristidis et al. 2015; Muda et al. 2023). However, in one research this hypothesis was confirmed, showing that people using their foreign language when solving moral dilemmas used less affect-rich vocabulary, and this change predicted their shift to more outcome-based responding (Kyriakou et al. 2022). Notably, this hypothesis was also criticized for using proxies for emotions, lacking a generalizable theory of emotion, and unclear baseline levels of neutrality (McFarlane and Cipolletti Perez 2020).

The second explanation—greater willingness to reflect when using a foreign language—also failed to be consistently confirmed. For example, people performed equally well (or, rather, equally poor) in the CRT in their foreign as in their native language (Costa et al. 2014; Milczarski et al. 2022; Vega-Mendoza et al. 2021). Had they reflected more, they should have performed in this test better.

The third explanation, proposed by Hayakawa et al. (2016)—that people use more abstract construals to represent a problem—gained little attention and was never directly tested. The single study I am aware of showed the two effects—changes in the decision and more abstract construals—in two separate experiments, never evidencing a direct path between the two effects (Shin and Kim 2017). 

So, how does using a foreign language affect people’s decisions through metacognition? Some insight can be gained through our research on the bias blind spot (Niszczota et al. 2022). We asked people to rate how likely they (and regular people) are to fall into some of the decision traps. As the Bible warned, people tend to look at the speck of sawdust in someone’s brother’s eye and pay no attention to the plank in one’s own eye (*English Standard Version Bible* 2001, Mat. 7:3–5). As in past research on this issue (Pronin et al. 2002), our results showed that people indeed see much more bias in others than they see in themselves. To avoid the bias blind spot trap, one must recognize that they are just as susceptible to bias as any other person and must be aware of their own potential biases. This requires metacognition. We observed a decrease in the bias blind spot in people using their foreign language. So, maybe they gained some sort of additional metacognitive awareness when using their foreign language? I discuss this hypothesis in the subsequent section. 

## 6. Debiasing through Metacognition? 

The answer to the question from the section above can be “Yes”. It becomes more plausible when considered in the context of findings suggesting that being bilingual or multilingual can provide cognitive benefits, such as improved attention, memory, and problem-solving abilities, compared to being monolingual (Bialystok 1988). This (highly debated and criticized, see e.g., Paap 2019) bilingual advantage hypothesis suggests that the process of constantly switching between languages and adjusting to different linguistic contexts can lead to improved cognitive flexibility, which is the ability to adapt to new information and changing task demands^4^. Speculatively, the benefits of being bilingual may be more pronounced when using a foreign language, leading to greater cognitive control and flexibility, and resulting in more metacognitive awareness of one’s mental states. Therefore, the foreign language effect would be a special case where the bilingual advantage is especially visible through more efficient metacognition. The reduced bias blind spot fits this hypothesis well.

Given this research, it appears that using a foreign language helps people to better use their metacognition and show less bias. If this is a true and replicable effect, this suggests that metacognition can be experimentally boosted, opening new avenues for the debiasing research. We dedicated some more resources to studying this hypothesis, i.e., that bilinguals, especially when using their foreign language, decide better when to think and when it is enough to rely on their gut feelings. We tested this hypothesis in experiments on reasoning, where people are asked to evaluate whether a syllogism (constructed of two premises and a conclusion) is logically valid. A logically valid syllogism is one in which its conclusion is always true given the premises are true. However, people tend to solve this task differently than asked—they first look at the believability of a conclusion, displaying so-called belief bias (Evans et al. 1983). When the conclusion is believable, people adopt a liberal responding strategy, preferring to accept the syllogism as valid. Differently put, they prefer to err by accepting an invalid syllogism as valid rather than by rejecting a valid syllogism as invalid. When the conclusion is unbelievable, they adopt a conservative response strategy, preferring to rather err by rejecting a valid syllogism (Dube et al. 2010). To reason correctly, one needs to reflect on the logical structure of the syllogism and ignore the intuitively available judgment of its conclusions’ believability. To make this process efficient, rather than inspecting logical structure of all syllogisms, one should query their metacognition. More specifically, one has to detect that logic and intuition are in conflict, and dedicate additional cognitive resources to reflect only on those syllogisms where logic and intuition are incongruent (Ackerman and Thompson 2017).

Across three experiments, we asked participants to evaluate a handful of syllogisms, either in their native or foreign language (Białek et al. 2020). Contrary to what I expected, we found decreased performance in the foreign language condition. The critical aspect was the conflict detection, a metacognitive cue that the initial response may be incorrect and that further reflection is needed. We observed that confidence ratings were higher in correct no-conflict trials (e.g., a valid syllogism with a believable conclusion) compared to incorrect conflict trials (e.g., an invalid syllogism with a believable conclusion). This shows that despite that the decision to accept a syllogism as valid reflected its believability, our participants were more conflicted when the validity and believability clashed. This is evidence for them being (to some degree) metacognitively aware of the possible error. However, the critical insight of our research was that conflict detection was lower in the foreign language condition. In other words, bilinguals using their foreign language were less able to detect when they might be wrong. As a result, they failed to reflect when reflection was needed. 

This finding was surprising: it showed that using a foreign language can indeed affect metacognition, but it distorts rather than boosts its quality. This made me formulate a model in which people differently track intuitions produced in their foreign vs native language. For example, foreign-language intuitions could be deprived of their affective component and thus less salient than native-language intuitions. Thus, people’s decisions made in a foreign language could be less affected by these foreign-language intuitions, resulting in better performance in tasks where an intuitive response is always wrong. However, this does not mean the everyday decisions made in a foreign language will be any better. Intuitions are evolutionarily calibrated quite accurately and are very useful (Gigerenzer 2000; Heyes 2019). Having our intuitions attenuated can deprive us of a hunch about what to do and can result in reflecting on things that do not require it or failing to reflect when needed. The former type of metacognitive error—underthinking—can lead us to make little use of our cognitive capacities and likely lead to suboptimal choices. The problems with the latter type of metacognitive error—overthinking—can pose two risks: computational errors and inefficient use of cognitive resources. 

While this hypothesis appears plausible, it has not yet been tested empirically. Some indirect support for this hypothesis comes from the moral foreign language effect. In moral psychology, we study how people weigh two moral inclinations against each other—deontological reliance on moral rules (e.g., do not kill) versus utilitarian reliance on the outcomes of a decision (e.g., kill one person to save five other people) (Conway and Gawronski 2013). Both of these considerations are intuitive (Bago and De Neys 2018; Białek and De Neys 2016). When a moral problem is considered in a foreign language, deontology and utilitarianism both lose their salience (Białek et al. 2019; Hayakawa et al. 2017; Hennig and Hütter 2021; Muda et al. 2018). Thus, a moral problem becomes less of a moral one and more of a regular problem where human lives transcend into mere numbers. This also aligns with the attenuated intuitions hypothesis. However, direct experimental evidence for the attenuated metacognition hypothesis, which is currently absent, is needed.

## 7. Summary and Conclusions

In the above, across almost 6.5 thousand words and about 90 citations, I argued how metacognition is critical to good decision-making and reviewed some of the recent research on debiasing through metacognition. This lack of thinking about their thinking and decision processes can lead people to bad decisions. If one focuses on the goal, even if it is an undoubtedly good goal such as national security and prevention of terrorist attacks, a lack of awareness of one’s own biased thinking can lead to disastrous decisions, such as the Guantanamo Bay torture program by the US government. Oftentimes, to decide well is to stop and reconsider one’s own decision process. 

Much of the research on debiasing is unsuccessful in achieving its effects, the effects are short-lived and malleable, and their boundary conditions are not well understood (IJzerman et al. 2020). However, there is a light in the tunnel: some manipulations do work in some conditions. Yet, we are still far from understanding how to help people. It seems that neither repeatedly solving similar problems nor being provided with feedback on past performance has a lasting effect on human performance. Using a foreign language also does not robustly affect decision-making, and if it does, its results are not always beneficial. 

Most of the manipulations that do work, such as the Nobel Prize-winning nudge (Thaler and Sunstein 2008), assist people in making a specific decision but do not necessarily lead to better decisions in the long term; but see (Van Rookhuijzen et al. 2021). A nudge is a type of choice architecture that reframes a problem to encourage a particular choice while still allowing decision-makers the freedom to decide. For instance, individuals may be automatically enrolled in a retirement savings plan but can choose to opt out. This way, more people sign in compared to when given a free choice whether to sign in or not. While leaving the debate about its moral permissibility aside (Bovens 2009; Schmidt and Engelen 2020), because of its incidental nature, the nudge direction of interventions may not be ideal. In the retirement savings example, people may save more money, but their overall attitude towards saving or their willingness to save actively in other ways may remain unchanged. Instead, the end goal of debiasing should be long-term, unsupervised, uncoerced, and free decision-making that results in better wealth, health, and happiness. 

I believe that we can achieve this goal and help people make better decisions by encouraging them to reflect on their own thinking. We need more experimental work on metacognition, preferably through field studies that capture real behavioral data, to test whether people can be trained to be more aware of their own thought processes, have better calibrated confidence, and use their cognitive resources more efficiently. Potentially, learning a second language could also help, but we need longitudinal data to determine whether metacognition and foreign language proficiency are positively correlated.

As I argue, metacognition is to some extent malleable and can be changed when using a foreign language. Among other consequences, people using their foreign language are less attracted to intuitively appealing but incorrect responses. Had we dedicated more resources to understanding how the foreign language affects metacognition, we could identify the critical features of this process and find methods that have similar features but do not require knowledge of a foreign language.

## Data Availability

Not applicable.

## Notes

1 People engage in reflection not necessarily to challenge their own decision, but sometimes they reflect to find support for their initial choice and to counter arguments for alternative solutions (Haidt 2012; Kunda 1990; Mercier and Sperber 2011). Hence, reflection is not a panacea to decision-making problems.

2 Altough the original is the most popular version of the CRT, there are several newer versions of the test, and all of them consist of just several items (Primi et al. 2016; Sirota et al. 2021; Thomson and Oppenheimer 2016; Toplak et al. 2014). Of these, the version by Sirota et al. (2021) is claimed to measure cognitive reflection with little to no interference of numeracy, which is a common issue in other versions of the test.

3 Much of the foreign language effect research investigates moral judgments (Białek et al. 2019; Geipel et al. 2015a; Hayakawa et al. 2017). However, as it is challenging to establish which morality is correct or erroneous, discussing "improved performance" in this context is not feasible. Thus, I will not delve extensively into this issue in this essay.

4 As with many psychological observations, the bilingual advantage is also not a definitive effect, with it being only conditionally observed, e.g., in some tasks, or mostly in older adults (Ware et al. 2020). Additionally, some of the bilingual advantages can be rather a result of higher socioeconomic status (Morton and Harper 2007) or a cultural effect (Samuel et al. 2018).
